# Starch-Based Functional Films Enhanced with Bacterial Nanocellulose for Smart Packaging: Physicochemical Properties, pH Sensitivity and Colorimetric Response

**DOI:** 10.3390/polym16162259

**Published:** 2024-08-09

**Authors:** Sanja Mahović Poljaček, Tamara Tomašegović, Maja Strižić Jakovljević, Sonja Jamnicki Hanzer, Ivana Murković Steinberg, Iva Žuvić, Mirela Leskovac, Gregor Lavrič, Urška Kavčič, Igor Karlovits

**Affiliations:** 1 Faculty of Graphic Arts, University of Zagreb, Getaldićeva 2, 10000 Zagreb, Croatia; maja.strizic.jakovljevic@grf.unizg.hr (M.S.J.); sonja.jamnicki.hanzer@grf.unizg.hr (S.J.H.); 2 Faculty of Chemical Engineering and Technology, University of Zagreb, Trg Marka Marulića 19, 10000 Zagreb, Croatia; imurkov@fkit.unizg.hr (I.M.S.); izuvic@fkit.hr (I.Ž.); mlesko@fkit.unizg.hr (M.L.); 3 Pulp and Paper Institute, Bogišićeva ulica 8, 1000 Ljubljana, Slovenia; gregor.lavric@icp-lj.si (G.L.); urska.kavcic@icp-lj.si (U.K.); 4 Danfoss Trata d.o.o., Jožeta Jame 16, 1210 Šentvid, Slovenia; igor.karlovits@danfoss.com

**Keywords:** anthocyanins, bacterial nanocellulose, maize starch, potato starch, pH-sensing films, smart packaging

## Abstract

Starch-based pH-sensing films with bacterial nanocellulose (BNC) and red cabbage anthocyanins (RCA) as active components were investigated in this research. Their structural, physical, surface and colorimetric properties were analyzed, mainly as a function of BNC concentration. The aim of the research was to relate the changes in the intermolecular interactions between the components of the films (starch, anthocyanins and BNC) to the physical, surface and colorimetric properties that are important for the primary intended application of the produced films as pH indicators in smart packaging. The results showed that maize starch (MS) was more suitable as a matrix for the stabilization of anthocyanins compared to potato starch (PS). PS-based films showed a lower value of water contact angle than MS-based films, indicating stronger hydrophilicity. The swelling behavior results indicate that the concentrations of BNC in MS-based films (cca 10%) and the concentration of about 50% BNC in PS-based films are required if satisfactory properties of the indicator in terms of stability in a wet environment are to be achieved. The surface free energy results of PS-based films with BNC were between 62 and 68 mJ/m^2^ and with BNC and RCA between 64 and 68 mJ/m^2^; for MS-based films, the value was about 65 mJ/m^2^ for all samples with BNC and about 68 mJ/m^2^ for all samples with BNC and RCA. The visual color changes after immersion in different buffer solutions (pH 2.0–10.5) showed a gradual transition from red/pink to purple, blue and green for the observed samples. Films immersed in different buffers showed lower values of 2 to 10 lightness points (CIE L*) for PS-based films and 10 to 30 lightness points for MS-based films after the addition of BNC. The results of this research can make an important contribution to defining the influence of intermolecular interactions and structural changes on the physical, surface and colorimetric properties of bio-based pH indicators used in smart packaging applications.

## 1. Introduction

The use of bio-based composites for packaging applications presents an innovative and sustainable approach to overcoming both environmental and economic challenges. By using various bio-based materials and converting agro-waste such as rice husks, wheat straw and sugar cane into packaging material, the packaging industry can significantly reduce its dependence on non-renewable sources such as petroleum-based plastics [1,2]. Not only does this mitigate the environmental impact of plastic pollution, but it also adds value to what would otherwise be disposed of as waste, opening up additional revenue streams and reducing the overall carbon footprint of packaging production. Furthermore, in some cases, the bio-based alternatives with recyclable and/or biodegradable properties can offer comparable properties in terms of durability and protection, making them a viable option for a wide range of packaging requirements. The adjusting of properties of biodegradable composites in packaging is revolutionizing the industry by addressing the critical environmental issues associated with conventional plastic waste. Unlike conventional plastics, which can persist over a long period of time, biodegradable packaging materials break down into harmless substances within a much shorter period of time, often aided by microbial activity [3,4,5,6]. This change not only mitigates the environmental impact, but also meets the increasing consumer demand for sustainable and environmentally friendly products.

As consumer demand for more sustainable materials and improved packaging solutions increases, the integration of agro-waste and bio-based composites is a promising step towards a more sustainable future. In addition, recent developments in the packaging industry, particularly in food packaging, offer packaging solutions with added value in the form of smart packaging solutions. By combining new technologies with conventional ones, and new materials with improved functionalities, smart packaging solutions offer various types of sensors and indicators that can communicate with the consumer and ensure the real-time monitoring of the packaged product. Their role is to display various alerts about changes in the packaging environment, indicate potential food quality issues and quickly inform the consumer about the contamination or spoilage of the packaged product [7,8,9,10]. As they are placed inside the packaging, they should be made of a material that meets the requirements of the European Parliament Framework Regulation (EC) No 1935/2004, Article 4 special requirements for active and intelligent materials and articles [11].

In the last decade, a significant innovation has been introduced in the production of bio-based films that can be used for smart packaging applications. Among the various materials, chitosan and starch composites, which can be derived from renewable sources, are the most widely used. Their main advantages are high availability, annual renewability, inherent biodegradability and good film-forming properties [12,13,14,15,16,17]. The main sources of starch used in various packaging applications are agricultural plants such as potatoes, rice, maize, wheat, tapioca and others [17,18]. In these plants, starch is formed as hydrophilic granules consisting of two glucose polymer components, the amorphous, branched amylopectin, the main polymer of starch, and the linear, crystalline amylose [17,18,19,20,21]. Amylopectin has two linked glucose chains, one α-1,4-glycosidic and one α-1,6-glycosidic, whose branched structure allows adjacent chains to form double helixes that assemble into crystalline lamellae, while branching points are located in amorphous lamellae. Amylose is a predominantly linear molecule consisting mainly of α-1–4-linked d-glucopyranose with a few α-1–6 branches. The proportion of amylopectin and amylose varies in the various sources of starch sources; the amylopectin content varies between 65 and 90% (*w*/*w*) and the amylose content between 10 and 35% (*w*/*w*) [19,20,21,22], but there are many exceptions [23]. Their content has a direct effect on the starch structure, the size of the starch granules and their film-forming properties, which are of great importance for the production of starch-based films that can be used in smart packaging.

In particular, starch-based films that can be used for the production of indicators in responsive packaging systems consist of a complex matrix made by incorporating different bio-based ingredients into the manufactured film, as well as natural pigments that are typically added to create a visual color response to pH changes in the packaging environment. By incorporating these natural pigments that respond to pH changes into starch-based films, it is possible to produce a pH-responsive sensor/indicator that can alert the consumer to certain food quality issues [10,14,23,24,25].

One of the most common natural pigments used for these applications as pH-sensing elements are anthocyanins, pigments found in red cabbage, blueberries, raspberries, grapes and other plants, as they change color in response to different pH levels. This type of integration of pH-sensitive elements into starch-based films not only adds value to the packaging, but is also in line with the principles of sustainability and biodegradability that apply to starch-based materials. As they change color depending on pH, they can be used for safety and quality assessment of foods that are mainly composed of proteins (e.g., chicken, beef or fish), where the decomposing proteins generate alkaline volatile nitrogen compounds that lead to pH changes on the food surface [10,12,26,27].

Another component used in the production of bio-based films and in the immobilization of natural pigments in responsive packaging systems is bacterial nanocellulose (BNC). It is used in bio-based smart packaging due to the positive physical and mechanical properties of the films produced and its excellent barrier properties. In combination with different active compounds such as anthocyanins, which are sensitive to pH, light, heat and oxidation, BNC has proven to be useful in protecting anthocyanins from degradation processes and positively influencing their stability [28,29,30,31,32,33].

Although the development of pH indicators that can be used in smart packaging has been extensively studied, the film formation process using different types of starch as well as the immobilization of anthocyanins using a BNC have not been thoroughly explored. The aim of this study was to produce a pH-responsive indicator for use in smart packaging by immobilizing anthocyanins in starch-based films. Furthermore, the influence of the addition of BNC on the structural changes and intermolecular interactions in the produced pH-sensing films was analyzed. The anthocyanins were extracted from outer leaf debris of red cabbage, and the polymeric matrix based on potato and maize starch, without and with the addition of BNC, was used for film production. A characterization of the anthocyanins extracted from red cabbage was carried out. Surface, chemical and colorimetric properties were measured on the films produced. The produced films were immersed in different pH buffers to observe and determine the visual change of the produced films. The adhesion parameters between the films and commercially available packaging material for raw meat were calculated to determine the adhesion possibilities when applying the produced starch-based films to the commercial packaging material. To our knowledge, the production and characterization of pH-sensing films from potato and maize starch with the addition of BNC and anthocyanins extracted from red cabbage has not yet been adequately reported. This research can make an important contribution to defining the intermolecular interactions and surface and colorimetric properties of bio-based pH indicators that can be used in smart packaging applications.

## 2. Materials and Methods

### 2.1. Materials, Extraction and Film Preparation

The polymeric matrix for production of a film-forming solution consisted of a potato starch (extra pure, CAS: 9005-25-8) (Carl Roth, Karlsruhe, Germany), maize starch (extra pure, CAS: 9005-25-8), distilled water, glycerol (purity 99.5%, CAS: 56-81-5) and glacial acetic acid (CAS: 64-19-7) (1% *v*/*v*) in different concentrations. Fresh red cabbage (*Brassica oleracea* L.) for extraction of anthocyanins was supplied from the local market.

Bacterial nanocellulose (BNC) was produced from the cellulose-rich biofilm formed after acetic fermentation of apple juice according to the method described in [34]. The prepared BNC was obtained in a suspension containing 0.15% of the dry matter of the BNC. The calculation of the dry matter content in the BNC suspension was 0.15%. It was carried out according to [35]. BNC suspension was isolated at a concentration of 10%, 30% and 50% (*v*/*v*) for the preparation of films.

Commercially available pH buffers pH 2.0–pH 6.0 [C_6_H_8_O_7_ (1-hydrate), NaOH, HCl] (Gram-Mol d.o.o., Zagreb, Croatia) and additionally prepared buffers pH 7–8.05 [Na_2_HPO_4_, C_6_H_8_O_7_] and pH 9.17–10.5 [Na_2_CO_3_-NaHCO_3_] were used.

#### 2.1.1. Extraction of Anthocyanins

The anthocyanins were extracted from the outer leaf debris of red cabbage. Red cabbage is known as a source of natural anthocyanins and contains more than thirty anthocyanin pigments in its extracts [12,36,37,38]. Red cabbage anthocyanins (RCA) are usually glucosylated cyanidins that are mono- or di-acylated with hydroxycinnamic acids. Acylated anthocyanins have improved heat and photostability properties compared to non-acylated anthocyanins and have been successfully used as a natural pH indicator [39,40]. For this research, the leaves of red cabbage were milled and mechanically blended with a 96% (*v*/*v*) ethanol solution (Pharmachem, Ljubljana, Slovenia) according to the modified method described in [41]. Extraction was carried out at 60 °C for 90 min, after which the samples were cooled and filtered using Whatman^®^ Quantitative Filter Paper, ashless, grade 40 (Cytiva, Malborough, MA, USA). The anthocyanin extracts were stored in the refrigerator before use.

#### 2.1.2. Film Preparation

The potato and maize starch films were made by mixing starch with other components in different concentrations. The first group of films contained potato starch (PS) and maize starch (MS) dissolved in distilled water and set on a temperature-controlled hotplate (Tehtnica, Rotamix 550 MMH, Domel, Železniki, Slovenia) with stirring (DLS Digital Overhead Stirrer, Velp Scientifica Srl, Usmate, Italy). Glycerol and acetic acid were added to the potato and maize solutions at a concentration of 20% (*v*/*v*) and 5% (*v*/*v*) for PS-based films and 15% (*v*/*v*) and 3% (*v*/*v*) for MS-based films, respectively. Specific glycerol and acetic acid concentrations for each starch-based film were chosen because our previous research has shown that different amounts of these components caused problems in the film formation and drying process. Since starch is composed of amylose and amylopectin molecules, acetic acid was added to the film-forming solutions to ensure that the branched amylopectin molecules break into straight-chain amylose molecules. Glycerol was then slowly added to the film-forming solution during the stirring process. Glycerol acts as a plasticizer and is incorporated into and between the polymer chains of the starch so that the hydrogen bond is broken and the chains spread apart. The incorporation of glycerol into the film-forming solutions increases the flexibility of the films produced and could influence other film properties such as water vapor, gas permeability, etc., in various packaging applications [42,43].

Different temperatures were set for the production of PS and MS film-forming solutions due to the different types of polymer matrices and the different processes that take place during film formation [44,45]. The PS film-forming solution was heated to 60 °C and the MS solution to 80 °C to ensure solubilization and the complete gelatinization process.

Starch film samples produced by dissolving PS and MS with the addition of glycerol and acetic acid were used as reference samples. To prepare a pH-sensing indicator for the detection of color changes in films, the anthocyanins were added to the PS film solution at a concentration of 12% (*v*/*v*) and to the MS film solution at a concentration of 14% (*v*/*v*). The concentration of anthocyanins was determined based on the previously performed experiments and their color response in different pH buffers. In addition, another series of samples were prepared containing different amounts of bacterial nanocellulose (BNC). The concentration of the BNC suspension was set at 10%, 30% and 50% (*v*/*v*) according to the total amount of film-forming solution. Depending on the type of starch used and the BNC concentration added, the films were designated PS or MS; 0BNC for films without BNC and 10BNC, 30BNC and 50BNC for different BNC concentrations.

A total of sixteen samples were produced, with eight samples based on PS starch and eight samples based on MS starch. The samples were designated as follows: PS_0 and MS_0 were designated as films without BNC and RCA; PS_0BNC_RCA and MS_0BNC_RCA were samples without BNC and with the addition of RCA; the samples named PS_10BNC_RCA contained 10% (*v*/*v*) BNC, the sample named PS_30BNC_RCA contained 30% (*v*/*v*) BNC, the sample named PS_50BNC_RCA contained 50% (*v*/*v*) BNC. The same designation was chosen for films based on MS starch. The prepared samples and their components are summarized in Table 1.

The production of each film took about 30 min with constant stirring. A certain volume of the film-forming solution was poured into a Petri dish to obtain a constant film thickness. The films were dried and stored in a ventilated climate chamber at 25 °C and 50% relative humidity (RH) for ten days.

### 2.2. Methods

#### 2.2.1. Determination of the Total Monomeric Anthocyanin Pigment Content

The total monomeric anthocyanin pigment content of red cabbage was determined using the pH differential method [46]. This method exploits the different absorbance exhibited by anthocyanins at pH 1.0 and pH 4.5 at a wavelength of approximately 520 nm. The change in absorbance is caused by structural transformation of the anthocyanins between the two pH values. A Shimadzu UV-1280 UV–Vis spectrophotometer (Shimadzu Scientific Instruments, Kyoto, Japan) was used for the measurements. The absorbance spectra of the samples were taken over the range from 250 to 750 nm, and the anthocyanin pigment concentration, expressed as cyanidin-3-glucoside equivalents, was calculated using Equation (1) [46]:

(1) $$Anth.pigment\left(cyanidin-3-glucoside\;equivalents,\frac{mg}{L}\right)=\frac{A\times MW\times DF\times 1000}{\varepsilon \times l}$$

where *A* = (*A*_520 nm_ − *A*_700 nm_)_at pH 1.0_ − (*A*_520 nm_− *A*_700 nm_)_at pH 4.5_; MW = 449.2 g moL^−1^ for cyanidin-3-glucoside; DF = dilution factor; *l* = path-length (cm) and *ε* = 26.900 molar extinction coefficient, in L/mol cm for cyanidin-3-glucoside and 10^3^ = factor for conversion from g to mg.

#### 2.2.2. FTIR–ATR Analysis

In this study, Fourier transform infrared spectroscopy–attenuated total reflectance (FTIR–ATR) of the prepared films was used to identify the changes in the interaction between the components in the PS and MS films, especially the interactions between the starch, BNC and anthocyanins used. FTIR–ATR analysis was performed using the IRAffinity-1 FTIR Spectrophotometer (Shimadzu, Kyoto, Japan). It contained ZnSe crystal type (index of refraction 2.4), and the resolution was set to 4 cm^−1^ with 15 scans. IR spectra were recorded in the range between 3600 and 800 cm^−1^, and the regions of interest (with detectable changes) were displayed.

#### 2.2.3. Swelling Experiments and Weight Loss Calculation

Film samples were immersed in water for periods up to 360 min. Normalized degree of swelling (M_t_) for control periods of 15, 30, 60, 90, 120, 180, 240, 300 and 360 min of immersion for each sample was calculated using Equation (2):

(2) $$M_{t}=\frac{m_{t}-m_{0}}{m_{0}}\times 100\%$$

where m_t_ stands for the weight of the swollen film at a time t, and m_0_ for the weight of the dry film before the immersion. It is important to emphasize that the swelling experiment was terminated for the samples which started to disintegrate, i.e., fall apart in water, and 360 min was enough for other samples to reach equilibrium. After the swelling experiment, samples were left in water until 24 h passed, transferred from the containers to glass substrate and then dried for 10 days at a temperature of 25 °C and relative humidity of 45 ± 5%. Then, they were weighed again using tare weight to determine the percentage of weight loss after the swelling in comparison to the dried sample before the water immersion.

#### 2.2.4. Surface Properties of Starch-Based Films

The surface properties of the films produced were analyzed by calculating the surface free energy (SFE) [47]. In a first step, the contact angles of probe liquids, demineralized water, glycerol and diiodomethane with known polar (γ_l_^P^) and dispersive (γ_l_^D^) components were measured. The total, dispersive and polar surface tension components of probe liquids, expressed in mJ/m^2^, were: water—72.8, 21.8 and 51.0; glycerol—64.0, 34.0 and 30.0 and diiodomethane—50.8, 50.8 and 0, respectively. The measurements were performed using a DataPhysics OCA 30 goniometer (DataPhysics Instruments GmbH, Filderstadt, Germany). The volume of the liquid drops was 1 µL in each case and the calculation was carried out using the sessile drop method. All measurements of the contact angle on the samples were performed at the same moment after the drop touched the sample surface and the average value of 10 measurements was calculated. The results of the water contact angles applied to the film surfaces were presented separately to determine the influence of added components in the films on their hydrophilic or hydrophobic properties.

The results of the contact angle measurements and the surface tension of all three probe liquids were used to calculate the SFE of the produced films according to the Owens–Wendt–Rabel–Kaelble method (OWRK) [48]. The calculation of SFE and its polar and dispersive components was performed to define surface changes in the produced films and to investigate their potential interactions—wettability and adhesion with other materials [48,49]. The adhesion parameters, the thermodynamic work of adhesion (W_12_), the spreading coefficient (S_12_) and the interfacial tension (γ_12_) were calculated [49]. For optimal adhesion, the value of the thermodynamic work of adhesion should be as high as possible, the spreading coefficient should be close to zero and the interfacial tension should be positive or equal to zero.

#### 2.2.5. Colorimetric and Visual Analysis of Starch-Based Films

The colorimetric properties of the films produced were measured using the Techkon SpectroDens spectrodensitometer (TECHKON GmbH, Königstein, Germany) (illuminant D50/2°, M1 filter). The measurements were carried out on dry film samples, on film samples that had not come into contact with different buffers and on film samples after immersion in different pH buffers for twenty minutes. A calibration was carried out on the integrated absolute white standard before the measurement. The CIE L*a*b* values were calculated and the relative CIE L*a*b* values (with paper as a white point) were displayed. L* stands for the lightness of the color, a* for the position between green and red and b* for the position between yellow and blue [50,51]. The results shown are the average values of ten measurements taken. Photographs were taken of films immersed in different buffer solutions with different pH values to observe the visual change in the films produced.

## 3. Results and Discussion

### 3.1. Determination of the Total Monomeric Anthocyanin Pigment Content

**Figure 1:**
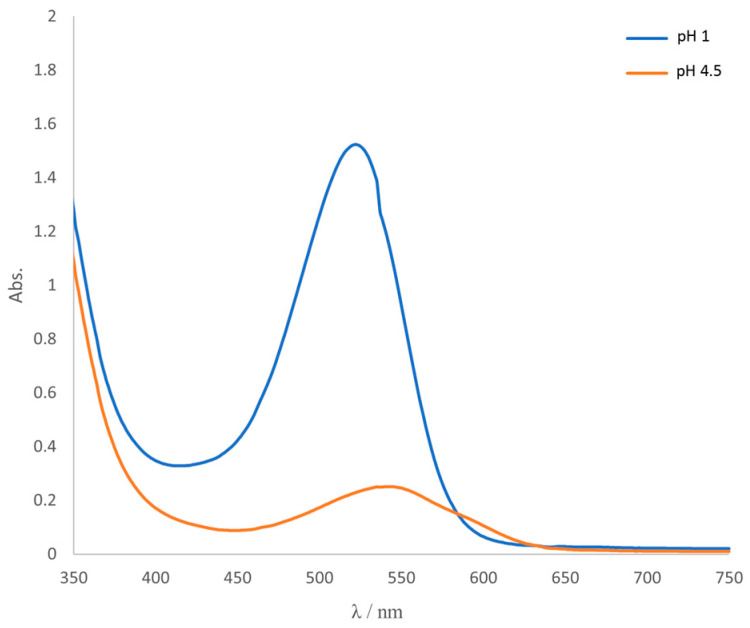
UV–Vis absorption spectra of red cabbage samples.

The UV–Vis absorption spectra of red cabbage extracts at pH values 1.0 and 4.5 are shown in Figure 1. Table 2 gives the absorbance values at 520 and 700 nm, respectively. According to calculation (Equation (1)), the anthocyanin concentration, expressed as mg of cyanidin-3-glucosides per 100 g of dry red cabbage leaves, has a value of 68.22.

### 3.2. FTIR–ATR Analysis of Prepared Films

**Figure 2:**
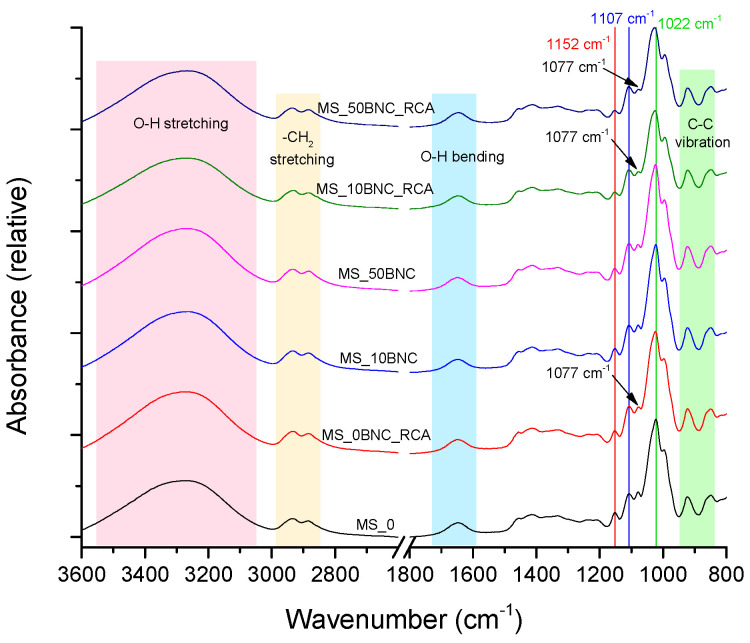
FTIR–ATR spectra of MS-based films without and with BNC and RCA.

**Figure 3:**
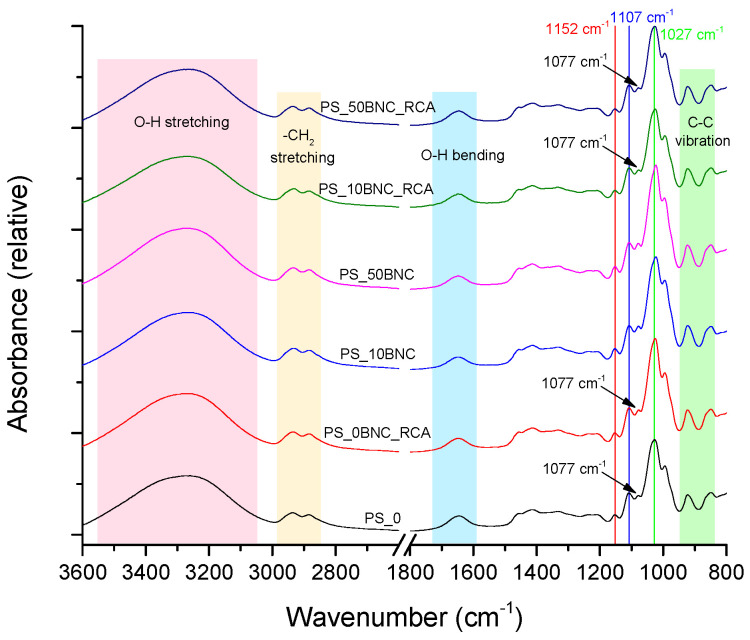
FTIR–ATR spectra of PS-based films without and with BNC and RCA.

Figure 2 and Figure 3 present the FTIR–ATR spectra of the selected starch-based films, without and with the addition of BNC and/or anthocyanins (RCA). In Figure 2, the characteristic bands and peaks for the maize-starch-based (MS) films can be observed, while Figure 3 presents the spectra of potato-starch-based (PS) films. Spectra for both films are similar, with MS-based films displaying more expressed changes related to the addition of the BNC and RCA compared to PS-based films.

The band between 3100 cm^−1^ and 3500 cm^−1^ peaking at around 3300 cm^−1^ belongs to O–H stretching vibration and is similar for all samples, regardless of BNC concentration or the addition of RCA to the starch. These O–H stretching vibrations are related to inter- and intra-molecular hydrogen bonds of hydrogen groups of starch [52,53]. The peaks between 2850 cm^−1^ and 2950 cm^−1^ are characteristic of C–H_2_ stretching in starch (and BNC) molecules [52,53]. Bands between 1630 cm^−1^–1690 cm^−1^ can be attributed to the H–O–H bending vibration of bound water in the films, which acts as a plasticizer, and to the O–H groups in cellulose [54,55]. The peak at 1152 cm^−1^ represents the anti-symmetric stretching of the C–O–C [56]. Two peaks in the region between 850 cm^−1^ and 950 cm^−1^, specifically at 925 cm^−1^ and 847 cm^−1^, are characteristic of the skeletal C–C vibration and α-configuration of carbon in the starch, respectively [57,58]. Listed bands and peaks are similar and present in the films based on both types of starches (Figure 2 and Figure 3).

Since the presented FTIR–ATR spectra of the films have a similar profile, without the formation of new peaks after adding BNC or RCA to the starches, it can be concluded that the interaction between the starch and BNC/RCA takes place, but without the creation of the chemical bonds [53]. Specifically, there are composition-related changes in the position or intensity of some peaks between 1022 cm^−1^ and 1107 cm^−1^ pointing to such interactions, observable in Figure 2. The peak at 1077 cm^−1^ presented a decreased intensity relative to the peak at 1107 cm^−1^ for all samples with added anthocyanins. The peak at 1022 cm^−1^ can be assigned to glucose in starch [53,59], and the peak at 1077 cm^−1^ represents specifically the crystalline regions of the starch [55]. This change in the peak intensity is prominent for the films with both BNC and RCA, and visible for the film with anthocyanins but no BNC, pointing to the interaction between anthocyanins and starch, which was improved after adding BNC [60]. The changes in the starch crystallinity may be due to the changes of the coupling forces between the crystalline and amorphous regions in the presence of anthocyanins, since the hydrophilic OH groups in anthocyanins might bind to starch and reduce its crystallinity [61].

Finally, the changes in FTIR–ATR spectra related to the intramolecular hydrogen bonding after the addition of BNC and anthocyanins to MS-based film can be detected in the peak at 1022 cm^−1^, which shifted to 1027 cm^−1^ for the samples with both BNC and RCA. This peak represents the C–OH bending vibrations, and the changes can be assigned to variations in the molecular environment of the primary hydroxyl group in amylose [62].

The FTIR–ATR spectra of the PS-based films (Figure 3) are similar to the spectra of MS-based films. However, it can be concluded that the interaction between PS and RCA or BNC is not as expressed as the interactions between MS and these components. Although there is a slight change in the intensity of the peak at 1077 cm^−1^ relative to the peak at 1107 cm^−1^ for the samples with both RCA and BNC, it cannot be exclusively related to their addition, since the similar relation between the peaks in question is present for the plain PS film. Furthermore, there is no shift of the peak from 1022 cm^−1^ to 1027 cm^−1^ when RCA or BNC are added to the PS-based film—the peak is at 1027 cm^−1^ for all films. This difference between the MS-based films’ and PS-based films’ FTIR–ATR spectra can be attributed to differences in the starch structural properties, the content of amylose and the behavior of the components in the starch at elevated temperatures during the film preparation [63,64].

### 3.3. Water Contact Angle on Films

**Figure 4:**
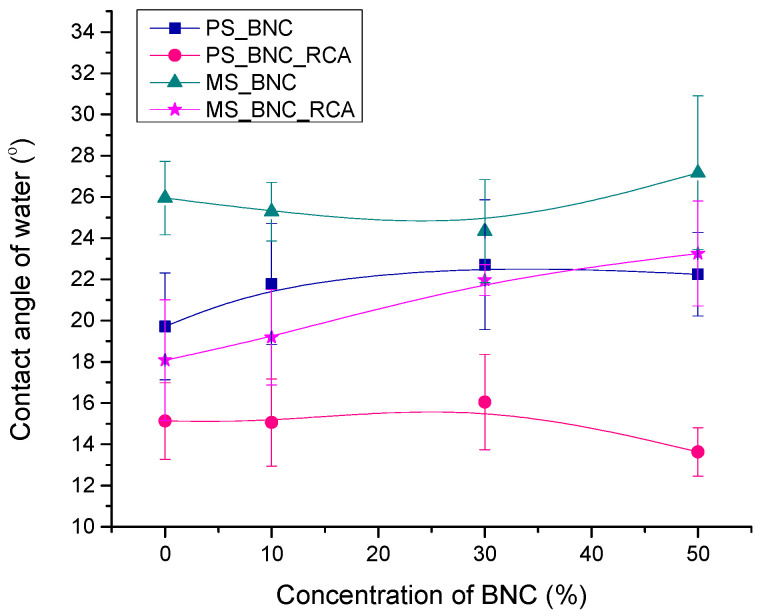
Water contact angle on starch-based films without and with RCA as a function of the BNC concentration.

The water contact angle can help in the characterization of the surface properties of the starch-based films with BNC and RCA by determining their hydrophilicity. Specifically, a lower water contact angle would point to the hydrophilic and hygroscopic nature of the produced films [33]. Figure 4 presents the water contact angle on films without and with RCA as a function of the BNC concentration.

In general, PS-based films displayed a lower water contact angle than MS-based films, pointing to more expressed hydrophilicity. Furthermore, both PS- and MS-based films with the addition of anthocyanins displayed lower values of water contact angle than films without them. Specifically, the minimal difference between the average water contact angle for PS-based films without and with the RCA is 5.09° (for the film without BNC), and the maximal difference is 9.12° (for the film with 50% BNC). The minimal difference between the average water contact angle for MS-based films without and with the RCA is 1.27° (for the film with 30% BNC), and the maximal difference is 6.87° (for the film without BNC). On MS-based films with a higher portion of BNC (30% and 50%), the effect of the RCA on the water contact angle is not significant, probably due to the better interactions between anthocyanins and starch/BNC via hydrogen bonding after the addition of BNC, as indicated by FTIR–ATR analysis. The general effect of the reduced water contact angle on films with added RCA compared to films without RCA can be attributed to their expressed hydrophilicity [65]. On PS-based films, the increase of the BNC concentration led to a mild decrease in the water contact angle, which could be attributed to the hydrophilic nature of BNC [55,66].

On the other hand, the addition of BNC to the film composition increased the water contact angle on MS-based films. Although cellulose consists of β-D-glucopyranose units which are responsible for the hydrophilic character of cellulose, electrostatic association and hydrogen bonding between BNC and starch could promote the hydrophobicity of the surface [33,67,68]. Different effects of BNC addition on the water contact angle of PS- and MS-based films could therefore be a result of their differences in structure, such as the size of the starch molecules, amylose content, etc. [17].

### 3.4. Swelling Dynamics and Weight Loss of the Films

**Figure 5:**
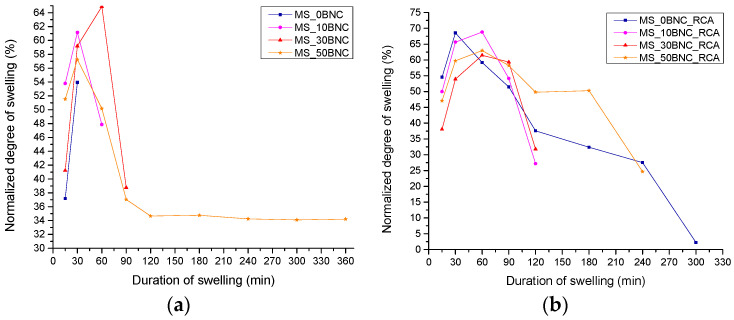
Swelling dynamics of MS-based films: (**a**) without RCA and (**b**) with RCA.

Figure 5 presents the normalized degree of swelling in water for MS-based films. Diagrams for PS-based films are not presented, since the partial disintegration of most PS-based films began to occur after only 15 min. Specifically, PS-based films that did have an initial period of swelling after immersion in water, before the beginning of the disintegration, were PS_0BNC, PS_10BNC and PS_50BNC, none of which contained anthocyanins. Their maximal normalized degree of swelling reached 20.22% for PS_0BNC, 26.78% for PS_10BNC and 32.28% for PS_50BNC after only 15 min. Then, normalized degree of swelling decreased to 10.84% for PS_0BNC, 20.90% for PS_10BNC and 16.86% for PS_50BNC after 60 min. After that, partial disintegration occurred followed by a weight loss compared to the dry sample. In other words, the addition of BNC and anthocyanins to PS-based films increased their compatibility with water in terms of the solubility parameters [69].

In Figure 5a, it can be seen that increasing the concentration of BNC to 50% in the films without anthocyanins led to the stabilization of normalized degree of swelling and to the preserved consistency of the film, i.e., no partial disintegration. On the other hand, the addition of anthocyanins to the films, especially to those with BNC (Figure 5b), resulted in decreased stability in water compared to a film with anthocyanins but without BNC and to the film with 50% BNC and no anthocyanins. A partial disintegration occurred for the MS-based films with anthocyanins and BNC after 120–240 min, when the swelling experiment was terminated. This behavior in water could be attributed to the polar nature of anthocyanins and the interaction between anthocyanins and BNC via hydrogen bonding, instead of the interaction of BNC with the starch that would lead to its stabilization in water [70].

**Figure 6:**
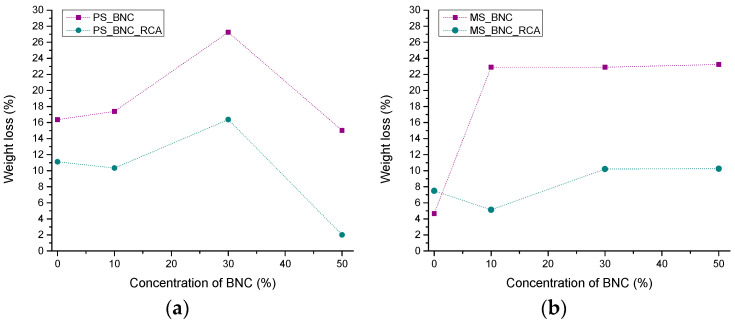
Weight loss of films after swelling: (**a**) PS-based films and (**b**) MS-based films.

Figure 6 presents the weight loss of the PS- and MS-based films after 24 h immersion in water and drying.

For both starches, it is visible that the addition of anthocyanins, regardless of the enhanced swelling in water, improved the stability of the films, i.e., led to decreased solubility in water. Maximal weight loss for the PS-based films with anthocyanins was 15.87% for PS_30BNC_RCA (a sample with the highest ratio of polar to dispersive SFE), and minimal weight loss of 1.51% was achieved for PS_50BNC_RCA (Figure 6a). A drop in the weight loss for PS_50BNC films compared to other PS-based films could be attributed both to the improved interaction between the components in the film and remaining bound water in the film structure. The addition of anthocyanins to the MS-based films resulted in a stable weight loss after the immersion in water for 24 h, which remained under 10% for all samples (Figure 6b) and pointed to the superiority of MS as a matrix for stabilization of anthocyanins compared to PS. The lowest weight loss of 4.63% was calculated for MS_10BNC_RCA film.

### 3.5. Surface Properties of the Films and Adhesion with Polypropylene

**Figure 7:**
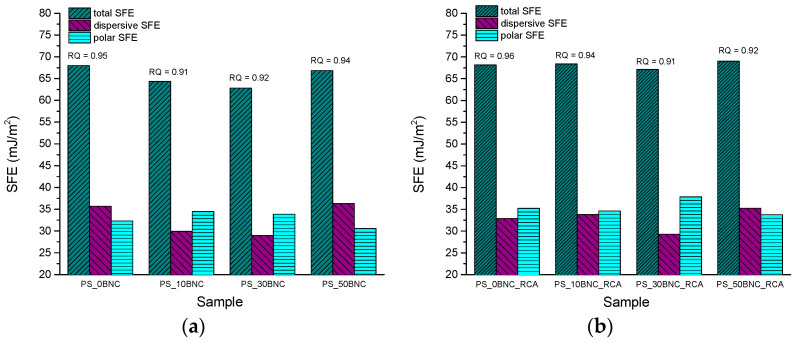
Surface free energy components of PS-based films: (**a**) with BNC and without RCA and (**b**) with BNC and RCA.

**Figure 8:**
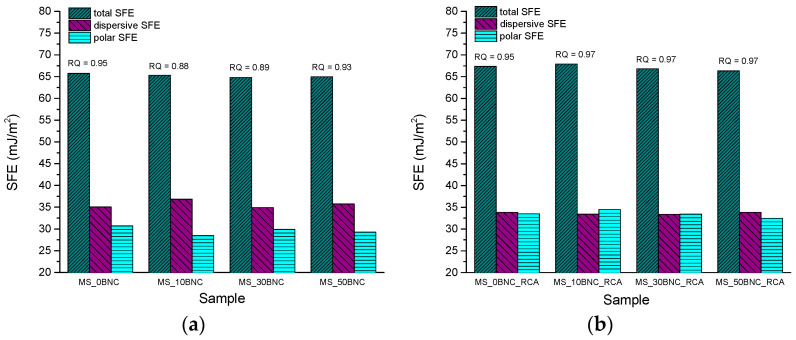
Surface free energy components of MS-based films: (**a**) with BNC and without RCA and (**b**) with BNC and RCA.

Results of the surface free energy (SFE) calculations for PS- and MS-based films are presented in Figure 7 and Figure 8, respectively. SFE and its dispersive and polar components are especially important in the packaging since they can predict the interactions with the specific substrate in contact with the pH-sensing films. Furthermore, SFE can be helpful in the discussion of other surface properties and swelling dynamics of the material, since any solvent initially needs to penetrate the material surface.

First, in Figure 7 and Figure 8, it is visible that all RQ values (error calculation used by DataPhysics SCA20 software) [71] are high. Specifically, the higher RQ, the better the linear fit and the accuracy of the SFE results obtained by OWRK model. RQ can provide information on the accuracy of the results when at least three referent liquids are used for the SFE calculations. RQ value should ideally be as close to 1 as possible [71]. When observing Figure 7, it can be noticed that the increased total SFE of all PS films after the addition of anthocyanins was primarily the result of the increase in polar SFE. This was expected because of a known polarity of anthocyanins.

For the films without anthocyanins (Figure 7a), polar SFE is higher than dispersive only for the samples with 10% and 30% BNC. Since BNC is of a polar nature [72,73], the increased share of the polar component of SFE after the BNC addition to the films is expected. The decreased polar SFE of the film with 50% BNC can be explained by electrostatic association and hydrogen bonding between BNC and starch that could promote the hydrophobicity of the surface [33,67,68]. This decrease of the polar SFE could therefore point to the improved interaction between PS and BNC, rather than BNC agglomerating in the film structure, which can happen if the starch and BNC are not compatible in terms of interfacial interactions [74,75].

After adding anthocyanins to the film composition (Figure 7b), the share of polar SFE increased for all samples. A dominant polar component of SFE should be noted because it can influence the application of the produced pH indicators in packaging, since many polymer materials for food packaging have a dominantly dispersive SFE.

Results of the surface free energy (SFE) calculation of MS-based films are presented in Figure 8. A similar trend of the increased polar SFE after the addition of anthocyanins as for PS-based films can be observed (Figure 8b). However, the share of polar SFE in MS-based films with BNC, but without anthocyanins (Figure 8a), decreased after the addition of BNC. This points to the interaction between the MS and BNC, which is superior to the BNC interaction with PS. This finding is aligned with the results of the swelling experiment, which showed that PS-based films started to disintegrate soon after immersion in water since apparently no sufficient interactions have been established between the starch, BNC and RCA to increase the stability of the films in water and improve their durability during the swelling experiment. A significant decrease in weight loss of PS-based films after the immersion in water for 24 h compared to other samples occurred only for the PS_50BNC sample. On the other hand, the addition of BNC improved the stability of the MS-based films in water during the immersion. When observing these results from a viewpoint of resource consumption, the recommendation can be made to use lower concentrations of BNC in MS-based pH indicators (cca. 10%), but a concentration of approx. 50% BNC is needed in PS-based films if satisfactory properties of the indicator in terms of stability in a wet environment are to be achieved.

Table 3 presents the calculated adhesion parameters, i.e., interfacial tension (γ_12_), work of adhesion (W_12_) and spreading coefficient (S_12_) between dry films and polypropylene substrate (PP), which is commonly used for packaging, especially for fresh food such as meat [76,77]. Calculated surface free energy components of PP substrate were 52.24 mJ/cm^2^, 31.74 mJ/cm^2^ and 19.51 mJ/cm^2^ for total, dispersive and polar components, respectively.

By observing Table 3, it can be concluded that the changes in interfacial properties between PP and the starch-based films with anthocyanins and BNC primarily depended on the addition of anthocyanins to the film-forming solutions. Although the differences in adhesion parameters among all samples are not generally high, it can be noticed that the samples with RCA displayed a slightly lower interfacial tension, higher work of adhesion and poorer spreading coefficient than films without RCA. MS films displayed marginally higher work of adhesion than PS films on PP substrate. However, it should be noted that the adhesion between the films and PP substrate is not optimal, which was expected since the films’ polar component of SFE has a significantly higher contribution to total SFE than that of PP. Specifically, interfacial tension, which should be as close to zero as possible for achieving the optimal adhesion, ranges from approx. 9 to 11 mJ/m^2^, and the spreading coefficient which should be positive for optimal adhesion is negative for all samples. This information is relevant from the viewpoint of packaging technology, since produced dry indicators, regardless of the high work of adhesion on the PP substrate, would not demonstrate optimal adhesion with this or other substrates of similar surface properties. It is noteworthy that polymer materials for food packaging usually have low surface free energy, with a particularly low polar component [78,79]. Therefore, in industrial production, proposed pH indicators should either be coated wet on the substrates, or attached to the substrate using a third component that would not influence the performance of the pH indicator.

### 3.6. Colorimetric Properties and Visual Analysis of Starch-Based Films in Varied-pH Environment

**Figure 9:**
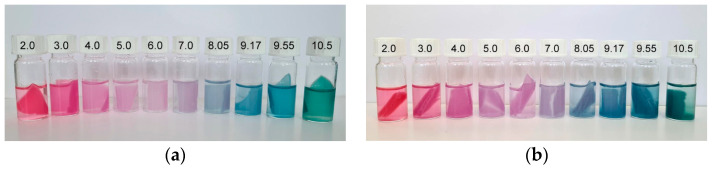
Visual color changes of films at different pH values (2.0–10.5): (**a**) PS_0BNC_RCA and (**b**) PS_50BNC_RCA.

**Figure 10:**
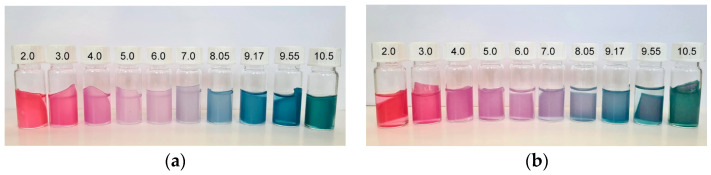
Visual color changes of films at different pH values (2.0–10.5): (**a**) MS_0BNC_RCA and (**b**) MS_50BNC_RCA.

Visual color changes of the chosen films after 20 min of immersion in buffer solutions (pH 2.0–10.5) are presented in Figure 9 and Figure 10. Figure 9a,b present the color changes of PS_0BNC_RCA and PS_50BNC_RCA films, respectively. A gradual transition from red/pink to purple, blue and green is visible for both samples. This transition is aligned with the results from previous research, where anthocyanins derived from red cabbage were investigated [80,81,82].

At low pH, the red-to-pink hue of the films was caused by the dominant form of the anthocyanin structure at that pH—flavylium cation. As the pH reached 5 and increased further, the red-to-pink hue turned purple and shifted towards blue/grey because of the development of a quinoidal base and anionic quinoidal base. Specifically, previously published research [83] reported that flavylium cations responsible for the red color were a primary form in anthocyanins at pH between 1 and 3, while deprotonation and hydration generated a carbinol pseudo base at pH 4–5. The quinoid base was formed at pH 7–8.

As the pH increased to 10.5, the color changed to green because anthocyanin started to degrade at high pH, causing the shift towards a yellow hue. This specific shift occurred because anthocyanins are susceptible to high pH, where the skeleton pyrylium ring opens and produces a chalcone structure due to the vulnerability to hydration and oxidation at the C2 position [81,84].

**Figure 11:**
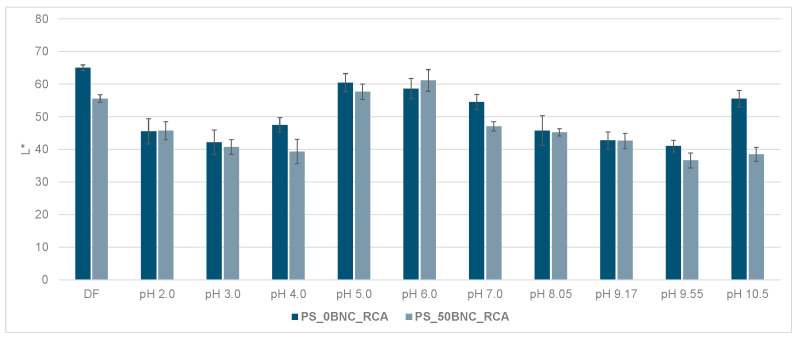
Lightness (L*) of PS_0BNC_RCA and PS_50BNC_RCA films at different pH values (2.0–10.5).

**Figure 12:**
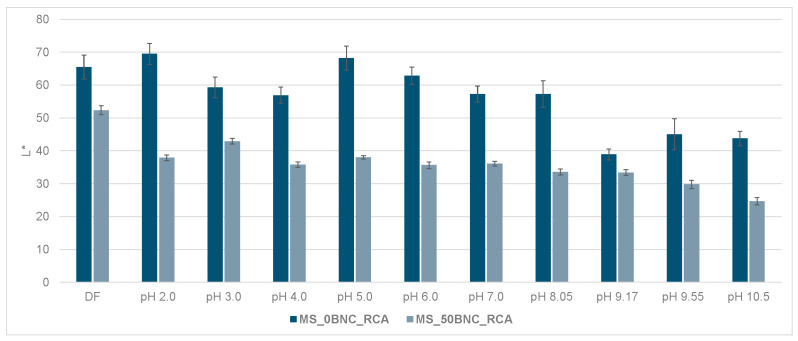
Lightness (L*) of MS_0BNC_RCA and MS_50BNC_RCA films at different pH values (2.0–10.5).

**Figure 13:**
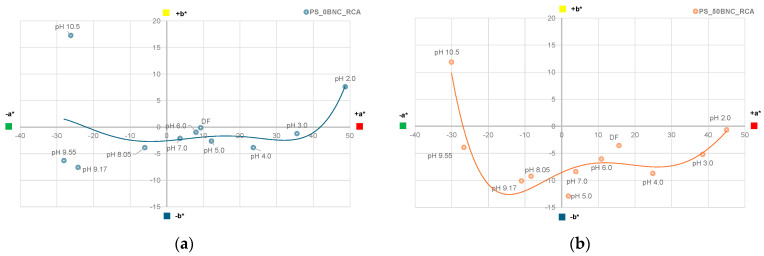
CIE a*/b* diagrams for films at different pH values (2.0–10.5): (**a**) PS_0BNC_RCA and (**b**) PS_50BNC_RCA.

**Figure 14:**
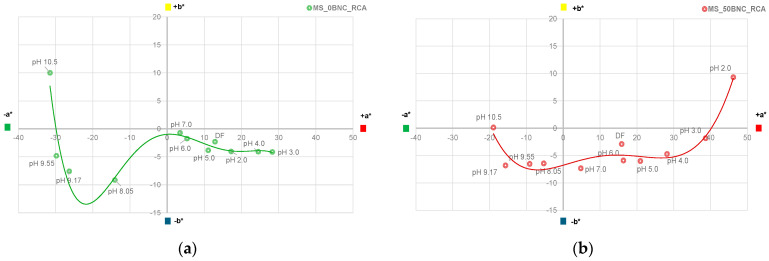
CIE a*/b* diagrams for films at different pH values (2.0–10.5): (**a**) MS_0BNC_RCA and (**b**) MS_50BNC_RCA.

The visual impression of the films in the environment of varied pH did not differ in terms of the hue when comparing PS-based films (Figure 9) with MS-based films (Figure 10), as expected. However, the visual impression of lightness differed for the films without and with BNC: films with BNC appeared darker. This visual impression can be attributed to the optical properties of BNC [85]. To analyze the color changes in the films numerically, their CIE L*a*b* values after immersion in buffer solutions were calculated and are presented in Figure 11, Figure 12, Figure 13 and Figure 14.

The decrease in the lightness of the samples with 50% BNC can be related to the optical properties of BNC and possibly the swelling dynamics of the films during the 20 min in the buffer solutions. All PS-based films and MS-based films without BNC, as well as the MS-based films with 10% of BNC, displayed faster and more expressed initial swelling in water than the MS-based films with a higher ratio of BNC (30% and 50%, visible in Figure 5b). Considering the composition of the buffer solutions and the methodology of the relative CIE L*a*b* measurements, the lower L* value as a direct result of the lower film thickness due to lower swelling could be expected. Furthermore, BNC by itself is not optically transparent [85] and certainly influenced the obtained L* values.

Figure 13 displays the changes in CIE a* and b* values of PS-based films, while Figure 14 displays the changes in MS-based films. To highlight and compare the trends of the CIE a*/b* shifts in different pH of the films’ environment, a polynomial trendline of fifth degree was added to the plots. Point “DF” stands for the “dry film” before the immersion in buffers.

The changes in a* and b* values of PS-based films are influenced by the addition of BNC mostly in the pH range between cca. 4 and 8 (Figure 13). The films with 50% BNC in this range had a more pronounced shift to blue hues (−b* values), pointing to the structural form of the anionic quinoidal base. This is significant since the degradation process occurs essentially from the anionic quinoidal base [86,87]. The points in the CIE a*/b* plots representing the pH 10.5 displayed a prominent shift towards +b* values (especially for the PS_0BNC_RCA sample), pointing to the chalcone structure and indicating the start of the anthocyanin degradation.

The changes in CIE a* and b* values of MS-based films are visibly influenced by the addition of BNC in the pH range between cca. 7 and 10.5 (Figure 14). Compared to the pH range of the BNC influence on a* and b* values in PS-based films, this is a significant shift. It could be concluded that BNC stabilized the anthocyanins in MS-based films at alkaline pH since the shift towards the hues characteristic of the anionic quinoidal base is not as prominent on the film samples with 50% BNC (Figure 14b). Finally, the point in the CIE a*/b* plot representing the pH 10.5 for MS_50BNC_RCA has a + b* value of only 0.13, pointing to the reduced presence of the chalcone structure [88] compared to the MS-based film without BNC.

Obtained results are aligned with the previous research, which demonstrated that BNC could play an important role in the stabilization and preservation of anthocyanins. Since BNC can form a network that physically protects anthocyanins from the degradative factors, it could extend their stability and lifespan. Furthermore, the addition of BNC to pH-sensing films in previous research created more vivid and stable colorimetric responses under varying conditions [89].

The results obtained in this research indicate that adding BNC to starch-based films, specifically when the films are MS-based, has a potentially significant advantage for applications in indicator systems and smart packaging. Furthermore, the results of the colorimetric measurements analyzed by observing the CIE lightness (L*) in a separate diagram and using CIE a*/b* plots could be helpful for the interpretation of the anthocyanin degradation dynamics in different media or in different conditions.

## 4. Conclusions

In this study, pH-sensitive starch-based films with bacterial nanocellulose (BNC) and red cabbage anthocyanins (RCA) as active components were investigated to observe their potential use as pH indicators in smart packaging. Two types of starch were used: potato starch (PS) and maize starch (MS).

The results showed that anthocyanins and BNC influence the crystallinity of the starch, and the swelling behavior and hydrophilicity of the produced films. The interactions between the main components of the films and the resulting changes in properties were significantly influenced by the BNC concentration and the type of starch.

The addition of anthocyanins to the MS-based films resulted in a stable weight loss after the 24 h swelling in water, which remained below 10%, indicating the superiority of maize starch as a matrix for the stabilization of anthocyanins compared to potato-starch-based films, which swelled excessively during the swelling experiment and started to disintegrate prematurely. The changes in the polar component of the surface free energy as a function of BNC concentration also indicated a better interaction between the maize starch and the bacterial nanocellulose. The changes in the a* and b* coordinates of the CIE L*a*b* color space of the PS-based films were influenced by the addition of BNC mainly in the pH range between about 4 and 8, while the addition of BNC altered the colorimetric response of the MS-based films mainly in the pH range between about 7 and 10.5. The colorimetric measurements indicate that BNC stabilizes the anthocyanins in the MS-based films at alkaline pH since the shift towards the hues characteristic of the anionic quinoidal base was not as prominent on the film samples with 50% BNC. In addition, colorimetric measurements showed that the addition of BNC to pH-sensing films resulted in more vibrant and stable colorimetric responses, which is a significant advantage for the application of starch-based films in smart packaging.

The results presented in this study show great potential for the use of starch-based films and bacterial nanocellulose with anthocyanins extracted from agro-waste residues of red cabbage for the production of bio-based pH indicators. The results demonstrate the possibility of their application in smart packaging or possibly even as an upgraded packaging material due to their relatively good mechanical properties. Further research, such as the measurement of mechanical properties, water barrier properties, density, antioxidant properties and other interactions with different packaging materials, is planned to further investigate the effectiveness of the presented pH indicators.

## Figures and Tables

**Table 1:** Starch-based samples produced in the research.

Sample	BNC Conc. (%)	Addition of RCA
PS_0BNC/MS_0BNC	0	/
PS_10BNC/MS_10BNC	10	/
PS_30BNC/MS_30BNC	30	/
PS_50BNC/MS_50BNC	50	/
PS_0BNC_RCA/MS_0BNC_RCA	0	RCA
PS_10BNC_RCA/MS_10BNC_RCA	10	RCA
PS_30BNC_RCA/MS_30BNC_RCA	30	RCA
PS_50BNC_RCA/MS_50BNC_RCA	50	RCA

**Table 2:** Absorbance values of red cabbage sample at 520 and 700 nm at pH values of 1.0 and 4.5.

Absorbance	pH 1.0	pH 4.5
*A*_520_	1.521	0.225
*A*_700_	0.023	0.011

**Table 3:** Adhesion parameters between the starch-based films and PP substrate.

Film Sample	*γ*_12_ (mJ/m^2^)	$W_{12}$ (mJ/m^2^)	$S_{12}$ (mJ/m^2^)
PS_0BNC	9.71	117.52	−18.48
PS_10BNC	10.12	113.47	−15.25
PS_30BNC	10.02	112.03	−13.61
PS_50BNC	9.37	116.71	−16.99
PS_0BNC_RCA	10.29	117.08	−19.19
PS_10BNC_RCA	10.16	117.47	−19.33
PS_30BNC_RCA	11.05	115.33	−18.97
PS_50BNC_RCA	10.02	118.25	−19.83
MS_0BNC	9.33	115.65	−15.85
MS_10BNC	9.03	115.53	−15.13
MS_30BNC	9.15	114.89	−14.73
MS_50BNC	9.08	115.14	−14.84
MS_0BNC_RCA	9.89	116.70	−18.04
MS_10BNC_RCA	10.13	117.02	−18.82
MS_30BNC_RCA	9.86	116.19	−17.451
MS_50BNC_RCA	9.66	115.91	−16.77

## Data Availability

The data presented in this study are available on request from the corresponding author due to privacy and contractual limitations.
